# Diabetic Retinopathy, a Comprehensive Overview on Pathophysiology and Relevant Experimental Models

**DOI:** 10.3390/ijms26209882

**Published:** 2025-10-11

**Authors:** Kate Gettinger, Deokho Lee, Yohei Tomita, Kazuno Negishi, Toshihide Kurihara

**Affiliations:** 1 Laboratory of Photobiology, Keio University School of Medicine, Tokyo 160-8582, Japandeokho.lee@keio.jp (D.L.); y.tomita@keio.jp (Y.T.); 2 Department of Ophthalmology, Keio University School of Medicine, Tokyo 160-8582, Japan; kazunonegishi@keio.jp; 3 The Korean Institute of Nutrition, Hallym University, Chuncheon 24252, Republic of Korea; 4 Laboratory of Chorioretinal Biology, Keio University School of Medicine, Tokyo 160-8582, Japan

**Keywords:** diabetes, diabetic retinopathy, streptozotocin, carotid artery occlusion, retinal ischemia, experimental models

## Abstract

Diabetic retinopathy (DR) is a serious complication of diabetes, leading to vision loss worldwide. The prevalence of DR has increased in recent decades. To understand the pathophysiology of DR, various experimental models have been developed and used. In this review article, we first outline what is currently known of the general pathology of DR, including the mechanisms involved in hyperglycemia, vascular dysfunction, retinal ischemia, retinal inflammation, and retinal degeneration. We next summarize various pathologies detected in experimental models in vivo, such as in chemically and genetically induced murine, rat, and monkey models, surgical methods in larger animals like cats, and a novel murine DR model using occlusion of the carotid artery under early diabetic conditions. A general overview of the in vitro models, including cell monocultures, co-cultures, and 3D models, is also provided. This current summary enables further research to obtain a more thorough understanding of DR pathogenesis and develop appropriate treatment measures.

## 1. Introduction

Diabetic retinopathy (DR), a common complication of diabetes mellitus (DM), is the leading cause of blindness among working-age adults [1]. According to the International Diabetes Federation, over 1 in 10 adults, or approximately half a billion people globally, suffer from diabetes [2]. However, this is expected to rise to nearly 800 million individuals by 2045 [3]. The prevalence of DR is currently estimated at over 100 million people and expected to rise to 161 million by 2045 [4]. The increase in DR is occurring in both high-income as well as low- and middle-income countries, but it is the low- and middle-income countries which will likely bear the greater burden of disease. The highest prevalence of DR is found in Africa, with North America and the Caribbean as the next highest prevalence rate [4]. Frequently identified risk factors for DR include poor blood glucose control, age, hypertension, and having DM for longer periods of time [5,6,7]. Somewhat paradoxically, DR risk has also been associated with insulin use and improved blood glucose control [8,9]. In a Chinese population with type 2 DM, the risk of DR increased with insulin therapy, possibly due to insulin’s impact on retinal microvasculature [10]. In a Korean population, insulin use, having DM for 10 years or longer, and higher systolic blood pressure were similarly identified as risk factors [11]. Treatment with glucagon-like peptide 1 receptor agonists in type 2 diabetics has also shown increased DR progression [12,13]. When considering that common treatments for DM may elevate the risk of DR, it becomes evident that its incidence and severity will continue to rise if we fail to understand its underlying mechanisms. Globally, there are about 7 ophthalmologists per 1000 vision-threatening DR patients, with Europe having the highest density while North America, North Africa, and the Middle East having the lowest density [14]. Diagnosing and treating DR remains a challenge, and unless we develop a better understanding of its pathogenesis, we will be unable to establish suitable treatments to stem the tide of this disease.

From what we do know, the pathology of DR is related to much more than just hyperglycemia. In fact, its complexity is in part why we are still unable to develop appropriate interventions. At its simplest, DR is characterized by its effect on the microvasculature within the eye. This can include neovascularization, ischemia, and changes in permeability, which in turn can lead to macular edema [15]. However, loss of retinal ganglia and axons has also recently been identified as an early change in DR and might even be the actual cause of it [16,17]. If neurovascular degeneration precedes angiogenic changes, this might explain why many screening methods for DR, which are based on vascular changes, could potentially delay diagnosis and treatment [15]. While control of blood glucose levels remains the mainstay of preventative treatment for DR and diabetic macular edema (DME), severe cases require interventions such as laser, anti-vascular endothelial growth factor (VEGF) treatment, steroid therapy, and vitrectomy [18]. However, as the efficacy of these treatments can vary widely, more research needs to be performed to establish a comprehensive understanding of DR pathology. As such, effective experimental animal models of DR are needed.

Currently, a number of animal models exist for studying DR, but each has its advantages and disadvantages. Chemically induced models, such as via streptozotocin (STZ) and alloxan, are quite popular for their convenience and low cost but have varying success rates, do not fully replicate the human condition, and can take significant amounts of time to develop the desired phenotypic changes. Combining the STZ mouse model with a new procedure known as unilateral common carotid artery occlusion (UCCAO) has shown promise as an accelerated model that can produce some of the ischemic changes typically lacking from STZ-only mice. Genetic models, including Akita mice, Akimba mice, db/db mice, and Goto–Kakizaki (GK) rats, are also able to replicate some aspects of the human condition of DR, such as neurodegenerative changes, but maintenance of some of these genetic lines can be expensive and tedious, with diabetes onset occasionally unpredictable. Surgical methods such as removing the pancreas can consistently produce a diabetic phenotype, but these are usually reserved for larger animals due to their technical complexity, and their ability to induce DR is debatable in some species.

In this review article, we summarize the currently used experimental models for DR and compare their characteristics. This summary enables further research to obtain more solid evidence of DR pathogenesis and develop novel treatment for DR.

## 2. Pathophysiology of DR

From what we currently know about DR at the time of this literature review from April to August 2025, it is a complex disease highly linked to metabolic pathways. One common pathological pathway is through the lack of insulin, which leads to increased levels of glucose distributed throughout the plasma, otherwise known as hyperglycemia [15]. This triggers several events that result in cell damage, vascular dysfunction, ischemia, and eventual degeneration.

Nearly all type 1 diabetics develop some form of DR within the first couple of decades of life, and two-thirds of all type 2 diabetics are afflicted [19]. In its early stages, patients may be completely asymptomatic of DR, but as time progresses, the condition can worsen, resulting in impaired vision due to edema and retinal hemorrhages [20]. From a clinical standpoint, DR can be divided into proliferative diabetic retinopathy (PDR) and non-proliferative diabetic retinopathy (NPDR). NPDR is generally considered the less severe, early stage of DR, where vascular permeability increases and capillary occlusion occurs [21]; it is typified by intraretinal microvascular complications [22]. It can be divided into mild, moderate, and severe categories based on retinal changes. Mild NPDR demonstrates microaneurysms only, while moderate NPDR includes eyes that show more findings than simply microaneurysms but are not severe enough to qualify as severe NPDR [23]. Severe NPDR consists of any of the following findings: more than 20 intraretinal hemorrhages throughout all four quadrants of the retina; venous beading in at least two quadrants; and/or intraretinal microvascular abnormalities in at least one quadrant and no signs of PDR [23]. Even with tight glucose control and a healthy diet, diabetic patients may still show signs of NPDR [24]. While NPDR may have no subjective symptoms, objective findings may include microaneurysms, hemorrhages of varying size, and hard exudates within the retina [21]. PDR demonstrates neovascularization in response to chronic retinal hypoxia, which can lead to vision-threatening microaneurysms and hemorrhages. When these fragile new blood vessels leak into retinal tissues, scar tissue can form, permanently impairing vision and increasing the risk of tractional retinal detachment. The neovascularization of PDR can occur in the iris, the angle, at the optic disc, and in the retina [25]. PDR can also include vitreous and pre-retinal hemorrhages. Clinically, PDR is graded on a scale of early, high-risk, and advanced based on the amount of neovascularization and whether DME is present [23]. Recent investigations have revealed that acidic proteins rich in cysteine (SPARC) are elevated in patients with PDR, and a subretinal injection of recombinant SPARC adenovirus into rats resulted in a PDR-like phenotype [26]. In addition, PDR patients have also demonstrated increased IL-8 levels in the vitreous [27]. mRNA sequencing revealed overrepresentation of pro-angiogenic processes in fibrovascular tissue from PDR patients, suggesting that this may explain why traditional intravitreal anti-angiogenic injections only have a transient benefit for PDR [28]. In addition, there is an increase in genes related to epithelial-to-mesenchymal transition, wound healing, inflammation, fibrosis, and extracellular matrix formation, indicating the complex nature of PDR [28]. The fibrovascular membranes formed as a consequence of PDR pose a definite threat to vision due to macular tractional retinal detachment [29]. Without proper intervention and treatment, PDR can lead to significant and permanent vision loss.

**Figure 1:**
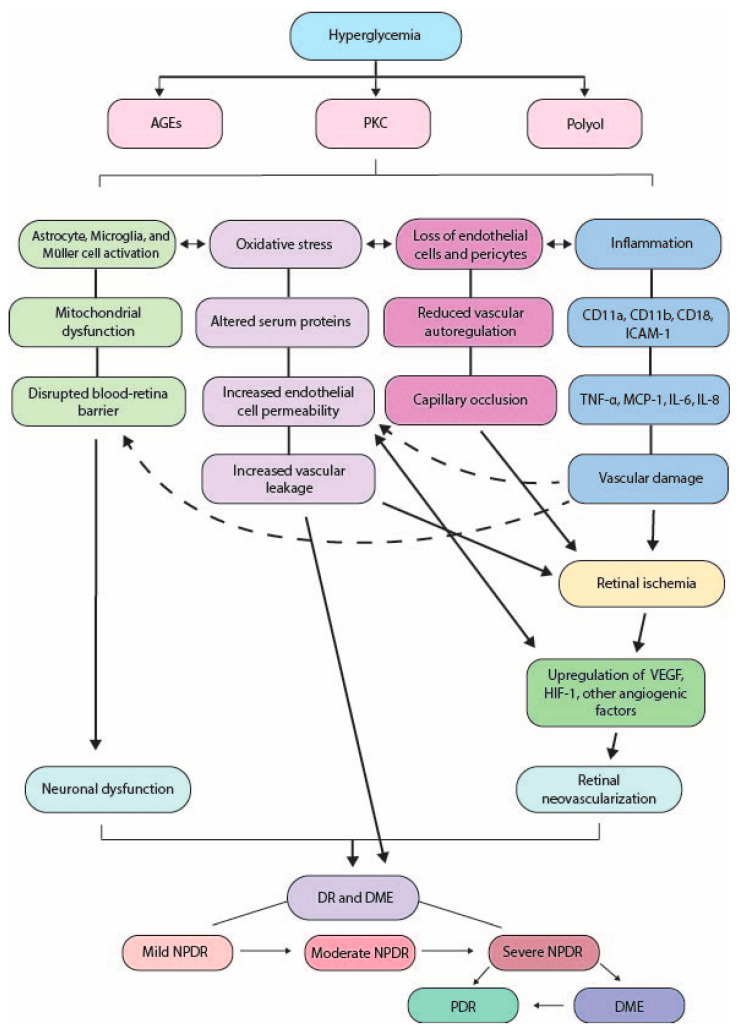
Summary of diabetes pathology. AGEs: advanced glycation end products; PKC: protein kinase C; ICAM-1: intercellular adhesion molecule-1; TNF-α: tumor necrosis factor alpha; MCP-1: monocyte chemoattractant protein-1; IL-: interleukin-; VEGF: vascular endothelial growth factor; HIF-1: hypoxia-inducible factor 1; DR: diabetic retinopathy; DME: diabetic macular edema; NPDR: non-proliferative diabetic retinopathy; PDR: proliferative diabetic retinopathy.

In this chapter, we will briefly summarize the current understanding of the pathophysiology of DR that leads to both NPDR and PDR. Figure 1 provides an overall summary.

### 2.1. Hyperglycemia

The persistent accumulation of glucose within the plasma results in the buildup of advanced glycation end products (AGEs) and general disruption of the polyol and protein kinase C (PKC) pathways.

Once formed, AGEs are essentially permanent [30]. The accumulation of AGEs has several known consequences. For example, AGEs promote the formation of reactive oxygen species (ROS), leading to oxidative stress in retinal cells [15,31]. The oxidative stress occurs due to altered serum proteins, disrupted formation of key structural proteins, or via buildup of endogenous byproducts of glucose metabolism [15]. Increased AGEs reduce mRNA expression of the protective, collagen-binding protein pigment epithelium-derived factor (PEDF) [15]. It is also thought that AGEs may increase retinal endothelial cell permeability, leading to vascular leakage [32]. In diabetic patients, there are higher concentrations of AGEs in ocular tissues [33], and the amount of AGEs in vitreous collagen has been correlated to DR severity [34].

Inflammatory damage occurs subsequent to activation of nicotinamide adenine dinucleotide phosphate (NADPH) oxidase and nuclear-factor B [15]. NADPH, along with sorbitol, is produced through the metabolic polyol pathway during hyperglycemia, making cells more susceptible to oxidative damage. During hyperglycemia, NADPH oxidase produces increased amounts of ROS [35], and these ROS can cause oxidative cell injury and death, as well as stimulate pathological processes of vascular remodeling [36]. Of the NADPH oxidase isoforms, NOX1, NOX2, and NOX4 have been identified as major contributors to DR [37].

Hyperglycemia also leads to activation of PKC and accumulation of diacylglycerol (DAG), both of which incite retinal vascular dysfunction and lead to DR [38].

### 2.2. Pericyte Loss and Vascular Dysfunction

Within DR, pericyte apoptosis [21], as well as loss of endothelial cells, and thickening of the basement membrane [39] disrupt the blood–retinal barrier (BRB), causing fluids to accumulate below and within the macula. In the early stages of DR, the diameter of retinal blood vessels increases, and autoregulation is reduced [40]. This is thought to be an attempt to respond to hyperglycemic changes and increased retinal metabolism [21].

Pericyte loss is an early-stage DR change, and their apoptosis may be due to overaction of the polyol pathway [41], as well as via upregulation of PKC, increased oxidative stress, and accumulation of AGEs [42]. As pericytes are lost, the structural integrity of vessel walls is compromised, resulting in microaneurysm formation. The loss of both pericytes and endothelial cells also leads to capillary occlusion, which results in retinal ischemia. This ischemia triggers upregulation of VEGF via the hypoxia-inducible factor 1 (HIF-1) signaling pathway [43]. VEGF is involved in a number of retinal vascular diseases, including DR [44]. Vascular permeability is increased by VEGF [45] and angiopoietins (Ang-1, Ang-2) [46]. It is becoming more evident that angiogenic factors like VEGF play an important role in PDR, and the balance between anti-angiogenic and angiogenic factors within the eye may be crucial in the treatment of PDR [47].

### 2.3. Retinal Ischemia and Inflammation

Inflammation is also a critical component of DR. In STZ-induced diabetic rats, retinal inflammation [48] and leukocyte entrapment [49] occur only a few days after diabetes onset. In diabetic patients with PDR, increased concentrations of inflammatory cytokines have been detected within the vitreous [50]. It is believed that upregulation of adhesion molecules drives the adhesion of leukocytes to endothelial cells, resulting in diabetic inflammatory damage. For example, upregulation of the surface integrin subunits CD11a, CD11b, and CD18 occurs in both diabetic rats [51] and diabetic humans [52]. In addition, diabetic rats demonstrate elevated levels of intercellular adhesion molecule-1 (ICAM-1), and blocking ICAM-1 results in reduced retinal inflammation and vascular leakage [48]. Similarly, STZ-induced mice with genetically induced deficiencies in CD18 and ICAM-1 show less inflammation and reduced vascular damage, leading to the suggestion that chronic subclinical inflammation may be responsible for many of the vascular changes seen in DR [53].

Chemokines, which regulate leukocyte adhesion and activation, also play an important role in DR-related inflammation. These chemokines are released by macrophages, including microglia, which gather around vessels in hypoxic areas of the retina [54,55]. Chemokines are elevated in the vitreous of diabetic patients [56], and the expression levels of tumor necrosis factor alpha (TNF-α), monocyte chemoattractant protein-1 (MCP-1), interleukin (IL)-6, and IL-8 have been shown to also correlate with DR severity [56,57]. The elevation of pro-angiogenic chemokines leads to changes in endothelial cells, which eventually result in neovascularization and vascular leakage [58].

Retinal ischemia onset in DR is relatively early and common [59], and most patients with diabetes will have some degree of retinal ischemia [60]. Even before the signs of DR appear clinically, patients with diabetes have shown reduced retinal blood flow [61], although this finding is controversial, as in early stages blood flow has also been shown to increase [62]. Capillary occlusion leads to retinal ischemia [63], which in turn can trigger some of the classic signs of DR including vascular leakage and neovascularization [60]. Retinal ischemia initiates many of the aforementioned inflammatory changes, such as VEGF expression and increased cytokine production, and therefore is usually seen in conjunction with inflammation [64,65]. Despite its indicated role in DR, a full understanding of ischemia’s influence is still relatively lacking, and more research is necessary.

### 2.4. Retinal Degeneration

Retinal glial cells are also affected by DR. Retinal glial cells include astrocytes, Müller cells, and microglia. Microglia are particularly vulnerable to early damage and usually initiate the cascade of neuroinflammation [66]. When astrocytes become activated in response to the metabolite imbalance brought about in diabetes, they secrete pro-inflammatory chemokines, including IL-6 and MCP-1 [66]. The secretion of TNF-α is also increased, which in turn promotes oxidative stress [67]. Meanwhile, Müller cells are activated by increased vitreous levels of HIF-1α and insulin-like growth factor 1 (IGF-1), and their activation stimulates the elevation of VEGF and basic fibroblast growth factor, which in turn promotes advancement of proliferative diabetic retinopathy [68]. It is also thought that the TNF-α and VEGF secreted by activated Müller cells disrupt the integrity of the blood–retina barrier [69,70].

There is some speculation that the retinal neurodegeneration present in DR might be an independent pathophysiology [21]. However, it is clear from diabetic experimental models that retinal neurodegeneration is a frequent finding in the disease. For example, in STZ rats, neural apoptosis begins as early as one month post-diabetes induction [71]. In diabetic human eyes, pro-apoptotic mitochondrial proteins are elevated [72], and high glucose levels in rat retinal Müller cells resulted in mitochondria dysfunction and apoptosis [73]. In both STZ-induced diabetic mice and diabetic humans, both the nerve fiber layer and inner retinal layers are thinned [74,75]. There have been some speculation that the retinal neurodegenerative changes precede the microvascular changes and thus may not be due to ischemic changes, but this remains controversial [74,76].

## 3. Experimental Models of DR

### 3.1. Streptozotocin

Streptozotocin (STZ) is commonly used to create animal experimental models of diabetes. STZ acts by accumulating within pancreatic β-cells and destroying them, which simulates type 1 diabetes [77].

Although STZ mice and rats are one of the most frequently used experimental diabetes models and generally thought to be relatively simple experimental models, there are some notable differences and inconsistencies. They exhibit variation in the diabetes-induced retinal metabolic dysregulation, which may in part be due to the activation of different metabolic components among species.

For example, STZ rats show a 26-fold increase in retinal sorbitol and a 4.7-fold increase in fructose, while STZ mice only demonstrate a 1.7-fold increase in retinal sorbitol and a 1.8-fold increase in fructose, although retinal glucose levels are comparable between the species [78]. Obrosova et al. also found a different distribution of antioxidant enzymes between STZ mice and rats. For example, superoxide dismutase is significantly higher in mouse retinas than in rat retinas, but other antioxidant enzymes were notably lower in murine retinas [78].

In STZ rats, retinal microglial cells are activated immediately following STZ-induction, and as early as four weeks after induction, STZ rats have notably denser concentration of microglia compared to controls [79]. ERG changes, however, typically take longer to manifest. While it has been shown that a-waves become delayed starting at three months after diabetes onset, it takes nine months before there is a significant difference in the b-wave latency [80]. Development of new blood vessels begins to occur around three months after diabetes onset [81], but it can take six to nine months before proliferative diabetic retinopathy phenotypes, including intraretinal hemorrhages and ischemic changes, can be observed [80]. STZ rats also demonstrate an increase in AGEs and receptor for advanced glycation end products (RAGE) in the retina [82] and plasma, liver, and kidneys [83], as well as an upregulation of TNF-α, IL-1, and IL-6, and overexpression of VEGF [82]. This increase in VEGF expression within the retina may be transient, with levels diminishing after four months [81].

In STZ mice, there is a similar pattern of retinal capillary damage and vascular leakage [84], as well as notable inflammatory changes [85,86,87]. However, these changes are notoriously inconsistent, and ischemic changes are often notably lacking in STZ murine models. Some of these variations may exist in part due to debate about the ideal dosage, time course of administration, and blood glucose cutoff values [88,89,90,91]. In addition, since STZ functions by destroying β-cells, it has been suggested that some instability may result from spontaneous β-cell regeneration and differences between mouse strains [92]. Gender also plays a role in the success rate of STZ induction, as female mice typically require a larger dose to induce diabetes [93]. For this reason, male mice are typically utilized.

While STZ mice have demonstrated inner retinal layer thinning [94] and ganglion cell loss [95], more severe phenotypes, including neovascularization [80] and oxidative stress [96], can take significant lengths of time to develop or are more controversial findings [97]. As such, STZ mouse and rat models are arguably limited to studying only the early phases of DR [92,98].

### 3.2. Alloxan

Similarly to STZ, alloxan is another commonly used chemical method for inducing experimental type 1 diabetes in mice and rats, although it has also been utilized in dogs, monkeys, rabbits, and cats [99,100]. It, too, acts as a toxic glucose analog and builds up in pancreatic β-cells, eventually leading to their death [101]. Alloxan also has some debate surrounding the ideal dosage and administration protocol [92]. Because of its notable inconsistencies and frequently abnormal findings, alloxan use has generally fallen out of favor in recent years [102,103].

Akin to STZ, phenotypic changes can take significant amounts of time to manifest in alloxan models. For example, one study demonstrated that alloxan rats can produce retinal pericyte ghosts and acellular capillaries, but only 18 months after diabetes induction, and there was no consistent evidence of microvascular abnormalities [104]. In another study of alloxan-induced diabetic rats, although fatty acid concentrations were shown to vary, there were no notable diabetic retinopathy changes observed [105]. Similarly, alloxan-induced diabetic swine, while showing an increase in Müller cell contraction, demonstrated no evident changes to the retina or blood vessels [106]. Due to these downsides, studies utilizing alloxan have become increasingly scarce in recent years.

### 3.3. Genetic Modulations

A number of animal genetic diabetic models exist for studying both type 1 and type 2 diabetes. Genetic models have the advantage of being more consistent and stable than chemically induced models, but they are often more expensive and take more maintenance. We will briefly outline some of the commonly used genetic models.

Akita mice (also referred to as Ins2^Akita^ mice) are one of the most frequently used genetic diabetic mouse models [107]. It was originally developed on the C57BL/6 background but are now commercially available with various combined genetic backgrounds [107]. It is a spontaneous type 1 diabetic model, wherein a point mutation in the insulin2 gene leads to pancreatic β-cell death [108], and hyperglycemia developing by two months of age [109]. At 12 weeks after diabetes onset, Akita mice demonstrate increased retinal vascular permeability, increased apoptosis, and alteration in astrocytes and microglia, as well as retinal thinning at 22 weeks [110]. It has also been reported that there is a significant reduction in RGC count at 22 weeks [111], but this finding has been debated [112]. Retinal neovascularization and a decrease in a- and b-wave ERG amplitudes have been noted eight to nine months after diabetes onset [109]. The Akita model is not without its limitations, however. For example, although the human condition of type 1 diabetes is regarded as a mostly autoimmune disease, the Akita model’s diabetes is not due to an autoimmune process, and thus this aspect of the disease is unable to be studied [98]. Akita mice also demonstrate β-cell regeneration, which is unlike the human condition and can complicate studies [113].

The Akimba mouse (Ins2^Akita^/VEGF^+/−^) is a cross between Akita and Kimba (VEGF^+/−^) mice, exhibiting hyperglycemia and retinal neovascularization [114]. At 12 weeks, Akimba mice have rod photoreceptor degeneration and inflammatory cells present, with evident changes in cell metabolism and ribosomal gene expression, gliosis, and immune system activation pathways [115]. Compared to Akita mice; however, Akimba mice show higher blood glucose levels from an earlier age, more severe photoreceptor cell loss, and greater retinal thinning with aging [114]. Clinical studies of photoreceptor cell loss in diabetic patients are scarce, but it has been suggested to play a role in DR development and is a potential area for future research efforts [116,117,118], further highlighting the usefulness of the Akimba model. Compared to Kimba mice strains, Akimba mice demonstrate more severe vascular changes, including neovascularization, capillary nonperfusion, fibrosis, and edema [114]. There is also increased macroglia and microglia activation, increased activation of perivascular macrophages, increased pro-inflammatory factors, and increased pro-angiogenic markers [119]. Overall, Akimba mice are capable of replicating many of the phenotypical changes seen in proliferative diabetic retinopathy. However, the severity of the phenotype can still vary. In addition, pre-retinal neovascularization is frequently not observed in Akimba mice, and there is a delay in ganglion cell loss [114]. Again, a major limitation of the Akimba model is that the neovascular changes are not due to hyperglycemia but rather due to the presence of the transgene hVEGF_165_ [114]. Because of this, the Akimba model is not suitable for etiological studies of diabetic retinopathy, as it differs from the true human condition.

The non-obese diabetic (NOD) mouse is a frequently utilized model for studying autoimmune changes in type 1 diabetes, despite having some notable differences from the human condition [77,120]. Similar to previously mentioned models, NOD mice develop diabetes through the destruction of pancreatic β-cells, with onset of diabetes occurring around 24–30 weeks of age [77]. Similar to human type 1 diabetic patients, NOD mice exhibit pancreas-specific antibodies and an increase in CD4^+^ and CD8^+^ T cells [121]. One disadvantage of NOD mice is that they require specific pathogen-free environments, as they are prone to immunomodulation from an expansive range of pathogens [77]. In addition, NOD mice have varying diabetes incidence rates depending on the gender, with females more likely to develop diabetes than males [77,120], and more severe perinsulitis and insulitis than in humans [120,122].

The db/db mouse model is a type 2 diabetic mouse model, showing diabetes-induced body weight gain, increased serum cholesterol, and increased serum glucose [123]. Db/db mice have a homozygous mutation in the leptin receptor gene, resulting in a lack of leptin, an appetite suppressant [124]. This leads to obesity and hyperglycemia. The db/db mouse model demonstrates reduced retinal ganglion cells [125] and a thinned inner limiting membrane (ILM) [126], as well as reduced endostatin [127]. They also exhibit increased caspase 3 (CAS-3) expression and matrix metalloproteinase 2 (MMP-2) and MMP-9 activation [123], which have pro-apoptotic roles, and increased expression of CD31, VEGF, and HIF-1α [126]. Both HIF-1α and VEGF have been positively correlated with the severity of retinal neovascularization [128]. In regard to the expression of RAGE, however, there seems to be conflicting findings [124,129]. While neurodegenerative changes have been confirmed via reduced ERG amplitudes and ganglion cell loss [125,130], these changes appear to occur before any observed vascular changes [130].

Goto–Kakizaki (GK) rats are the spontaneous rat model of non-obese type 2 diabetes. They are based on the Wistar rat background and developed by repeatedly inbreeding glucose-intolerant Wistar rats [98], with diabetes developing around 4–6 weeks of age [131]. Retinal angiogenesis sets in after about 6–7 months, accompanied by increased expression of angiogenesis factors such as VEGF, PDGF, MMP-2, MMP-9, and IGF-1 [132]. There has also been an observed increase in the endothelial/pericyte ratio beginning at eight months of age and continuing to increase over time [133]. At 12 months, there is a notable accumulation of microglia and macrophages within the subretinal space, as well as alterations to photoreceptor outer segments and vacuolization of RPE cells [134]. One arguable limitation of this model, however, is that early β cell destruction does not truly represent human type 2 diabetes [77].

### 3.4. Surgical Methods

One of the most common methods for surgically inducing diabetes is through removal of the pancreas, or a pancreatectomy. Removing the pancreas induces hyperglycemia and replicates type 1 diabetes. This model has been used for a variety of species but is mostly restricted to larger animals due to its technical complexity, including cats, dogs, and monkeys.

In cats, hyperglycemia develops as early as one week after pancreatectomy, and after three months, signs of DR such as capillary basement membrane thickening begin to appear [135]. Other signs of DR, including microaneurysms, capillary nonperfusion, and neovascularization, develop five years after surgery [136]. However, these long-term findings are not always consistent, and severity levels can vary [137].

In a study of rhesus monkeys, removal of the pancreas induced insulin-dependent diabetes but did not appear to result in DR findings, and even 15 years post-surgery, there were only mild blood–retinal barrier disruptions [138]. Within the same study, chemically induced or spontaneously hyperglycemic monkeys, in contrast, did demonstrate ischemic retinopathies such as cotton wool spots and were thus better for studying DR [138]. These animals showed areas of capillary dropout, arteriolar and venular occlusions, vascular leakage, and signs of macular atrophy. A number of studies utilizing spontaneous type 2 diabetic monkeys have exhibited findings such as retinal nerve fiber thinning [139], activation of AGE/RAGE [139], intraretinal hemorrhages [140,141], and retinal capillary nonperfusion [140]. In a recent study utilizing STZ-induced type 1 and high-fat-diet-induced type 2 DM rhesus monkeys, several early-stage DR neurodegenerative changes were observed [142]. As such, these spontaneous or chemically induced models are often preferred for studying DR in non-human primates. However, as it has been noted that progression to proliferative retinopathy and other advanced diabetic complications such as diabetic nephropathy are rarely observed in any rhesus monkey models, their use for studying advanced stages of the disease appears challenging [138,143].

While surgically induced diabetic dogs are used for diabetes research, and pancreatectomy in adult dogs for research purposes has been documented as early as 1922 [144], they are not frequently utilized for studying DR. Dogs with spontaneous, chemically induced, or diet-induced diabetes have been claimed to have the most similar retinal findings to human DR [145,146]. In fact, specifically galactose-fed dogs have been argued to be the closest representation of human diabetic retinal lesions [147]. As such, these models are usually favored over surgical interventions when it comes to studying DR.

### 3.5. Streptozotocin + UCCAO

Recently, a new mouse model of diabetic retinopathy was developed by combining STZ injection with a unilateral common carotid artery occlusion (UCCAO) procedure. As mentioned previously, one of the major limitations of the STZ model is that it often takes close to a year for significant phenotypes to develop, and most changes are non-ischemic [86,148,149]. The STZ model very rarely develops any proliferative DR changes [150].

Retinal ischemia has emerged as playing an important role in DR but is challenging to study in most established experimental diabetes models. Instead, retinal ischemia is typically studied using models of oxygen-induced retinopathy [151]. Recently, the UCCAO mouse model has been established as a stable, consistent model of retinal ischemic stress, demonstrating acute retinal gliosis, decreased retinal function, and neurodegeneration [152,153]. Since mice do not have a circle of Willis, as in humans, bilateral common carotid artery occlusion creates fatal brain ischemia, complicating efforts to study retinal ischemia [154]. However, UCCAO results in stable, observable retinal ischemic changes, although there is no diabetic component.

By performing UCCAO in STZ-induced diabetic mice, a DR phenotype with ischemic changes can be observed within a shorter time frame than in typical STZ-only studies [155]. At six weeks after diabetes onset, and at one day and one week after UCCAO was performed, there was an increase in retinal inflammatory cells, a significant decrease in capillary vessel diameter, and increases in the retinal mRNA expression of *Ccl2*, *Ccl12*, *Bnip3*, *Pdk1*, *Hsp25*, and *Vegfa* compared to controls and STZ-only mice [155]. As such, this model could serve as an accelerated model of DR that allows for the study of both inflammatory and ischemic vascular changes. However, more research needs to be carried out to fully establish this new model.

### 3.6. In Vitro

A major downside to animal studies, especially when studying treatments, is that the use of different species may lead to misleading or contradictory results. In vitro studies are able to fill this gap and help confirm the effectiveness of treatments in humans with more consistency and accuracy, and without ethical limitations. There are a number of in vitro models, from the simplest single-layer cell culture to complex organoids. Which model is utilized depends on factors such as the research goal and lab resources.

Cell monocultures, or cell cultures of a single type of cell, are perhaps the most straightforward and easiest, as well as being generally low cost and able to yield reproducible results [156]. For DR studies, they are usually made of either RPE or glial cells. Among the RPE cells, the mostly commonly used is ARPE-19 [157]. It was produced spontaneously from a 19-year-old donor and is useful when a high yield of cells is needed. However, culture conditions can vary the differentiation of ARPE-19, and care must be taken when using them to ensure compatibility with the desired study [157]. Alternatively, fetal human RPE cells (fhRPE) are also used in monolayer cell cultures, especially in studies where the barrier function of the cells is important [158]. While ARPE-19 cells are useful for representing diseased-state RPE, fhRPE cells represent normally functioning RPE, making both useful for RPE studies [158]. Glial cells are also frequently used in monolayer cell cultures to study the response of Muller cells, astrocytes, and microglia to diabetic stress factors. For example, the human retinal Muller cell line MIO-M1 can be used to study the regulatory roles of various signaling pathways [159]. The MIO-M1 cell line was derived from an adult human retina and maintains characteristics of neural stem cells [160].

Another commonly used cell line is the BV-2 cell line, which is an immortalized murine microglial cell line originally derived from C57BL/6 mice [161]. BV-2 cells have proven to mimic primary microglia in many ways, but differ in some aspects, such as the expression levels of TNF-α and IL-6 [162]. In regard to diabetic retinopathy studies, BV-2 cells have been used to study the microglial neuroinflammatory response to hypoxia [163,164].

Stem cell-derived models are also useful for studying diabetic retinopathy, especially when exploring regenerative treatment options. The two most used stem cells include human embryonic stem cells (hESCs) and human induced pluripotent stem cells (hiPSCs) [156]. hESCs, as the name suggests, are derived from embryos, but their ability to differentiate into various cell types is still being established, as appropriate culturing conditions have not been fully described [165]. hiPSCs, meanwhile, are differentiated cells that are shifted back into a pluripotent state and can differentiate into the ectoderm, endoderm, and mesoderm. For both hiPSCs and hESCs, the culture conditions can influence the development of cell lines into RPE cells and photoreceptors [166,167]. hiPSCs have also been used to generate diabetic retinopathy-specific RPE models [168,169]. A major advantage of using hiPSCs to establish RPE cells for study is that they maintain physiological and morphological attributes of human RPE while exhibiting similar functionality [170].

Co-cultures essentially have all the benefits of monolayer cell cultures, with the added advantage of being able to study the interactions and signaling pathways between cell types. When pericytes and endothelial cells are co-cultured, it allows for the study of microvasculature and the BRB. For example, Tarallo et al. used a retinal pericyte and endothelial cell co-culture exposed to high glucose to study diabetic retinal vascular damage [171].

In diabetic retinopathy, vascular abnormalities often lead to notable visual impairment, and so a large amount of focus has been given to endothelial cells and other vascular components. However, glial cells may play a role in the mechanisms driving these vascular changes. Wu et al. explored the effect of exposing a co-culture of microglial cells and endothelial cells to varying concentrations of glucose and demonstrated that microglial cells responded to high glucose levels. In addition, they also showed that microglia had an influence on drug efficacy, indicating how studying these cellular interactions are of critical importance when developing DR treatment strategies [172].

3D models are also useful in vitro models for studying diabetic retinopathy. While 2D models are the most frequently used, they are limited in regard to the complexity of the interactions that can be explored. Three-dimensional models allow for the study of the complex mechanisms involved in DR without the ethical constraints of animal models. For example, stem cells can be used to create 3D tissues that mimic real organs, resulting in what are known as organoids. hiPSCs and hESCs are frequently used to develop retinal organoids [156]. In a study by Gore et al., a 3D spheroid model using RF/6A choroid-retinal vascular endothelial cells was used to study retinal and choroidal pathological vascular changes in DR [173]. While these more complex models can be highly useful for studying particular aspects of DR, limitations in lab resources and skills may pose potential barriers to their use.

Table 1 offers a summary of all the experimental models of DR discussed above.

## 4. Recent Aspects of DR Research

Both in vivo and in vitro models have permitted great strides to be made in our understanding of the pathology of DR. Experiments involving well-established DM models, as well as attempts to create new and improved study models, continue to build upon our base of knowledge. These experimental models can prove useful as novel research aims are developed over the coming years. For example, some recent human epidemiological studies have provided surprising correlations that provoke new research paths requiring further exploration. For example, it has been suggested that myopia, or near-sightedness, may hold a protective effect against DR. Eyes with high myopia demonstrate lower incidence of PDR and NPDR [178]. Axial length has been shown to have a significant negative correlation with the severity of diabetic retinopathy [178,179,180,181,182]. One meta-analysis found that for every 1mm increase in axial length, there was a 23% reduction in the risk of DR and a 37% reduction in the risk of vision-threatening DR [183]. This reduced risk seems to be most highly related to the axial length, as some studies have demonstrated no relationship between diopter level and DR [180]. One study demonstrated that while men are more at risk of DR, this does not impact the reduced risk conferred by axial length [184]. However, there has been some conflicting findings among younger patient populations, where a longer axial length was linked to higher incidence of diabetic tractional retinal detachment [185]. In addition, while some research has suggested that increased HbA1c and blood glucose levels may contribute to myopia progression [186], another study demonstrated a cohort of type 2 diabetic patients undergoing tight glycemic control exhibited a negative relationship between HbA1c and choroidal thickness [187]. Utilizing animal models to investigate these relationships in more depth may help explain these findings.

It has also been shown that there is a positive correlation between metabolically induced hyperopic changes and blood glucose reduction in patients receiving glycemic control treatment for diabetes [188]. It has been speculated this is due to a change in the refractive index of cortical fibers of the lens due to hyperglycemia, and chronic hyperglycemia slowed axial length growth in alloxan-induced diabetic rabbits [189]. More experimental models to examine this relationship in greater detail may be warranted. Based on findings, future screening methods for DR risk in humans may include assessment of axial length and refraction status.

Although current treatments emphasize control of blood glucose, recent studies have indicated only about 11% of the variation in DR risk can be attributed to HbA1c levels and the duration of diabetes [24]. It has been suggested the remaining 89% may be due to varying environmental and genetic factors, biological mechanisms independent of glycemia, and glucose variability via oxidative stress, but these still have not been substantially described [24]. Recent research has attempted to identify other ocular and systemic biomarkers to help in the prediction of risk, as well as improve diagnostic rates. In comparison to the entire body, the retina represents a comparatively small portion of the total mass, and so any plasma-based biomarkers need to be highly specific [190]. Attempts at identifying local biomarkers, which would be more reliable, are complicated by the fact that obtaining samples of aqueous humor, vitreous, or retina is an invasive and potentially complicated procedure [190,191]. However, using biomarkers identified from tears has been suggested both for detection of diabetic risk and for other systemic diseases, and holds promise [192].

Recently, the neurodegenerative changes in diabetes have also been receiving attention. As mentioned previously, debate still exists about whether neurodegenerative changes precede vascular changes in DR. Polyneuropathy is significantly associated with both DR and nephropathy [193,194,195,196,197], and it has been suggested this relationship could be utilized to help with the prediction of complications. As there appear to be overlapping pathogenic pathways between DR and neuropathy, integrated management approaches would likely prove beneficial [194]. It has been suggested that dysregulation of growth factors, such as transforming growth factor-β1 and VEGF, are associated with DR and diabetic neuropathy and could serve as potential biomarkers to help triage both complications [198]. However, most attempts to identify biomarkers for predicting diabetic sensorimotor polyneuropathy have proven disappointing, with low predictive value and accuracy [199]. Finding one single biomarker to predict risk seems less likely, and instead it has been suggested that research should focus on identifying a strategy for interpreting a complex assortment of biomarker data. Artificial intelligence (AI) could be utilized to help identify patterns in biomarkers and improve predictive values [199].

There have also been recent attempts to utilize AI to improve diagnosis and risk prediction for diabetes-related ocular complications. Using clinical biomarkers such as optical coherence tomography (OCT) scans and fundus photographs, in addition to basic demographic information and clinical data, with AI algorithms can improve automated diagnosis of DR [200,201], although further refinement is needed. These algorithms may eventually help improve screening and detection of diabetic risk, especially in underserved populations without regular access to ophthalmology clinics.

## 5. Conclusions

Diabetic retinopathy is a complex, multifaceted disease. Despite its widespread prevalence, we still do not have a solid understanding of the pathology of this disease and thus are unable to develop appropriate treatments. A major component to unraveling the mystery of DR is the use of experimental models. Each model has advantages and disadvantages, and there is no single model that can capture the full spectrum of the human DR phenotype. As such, it is better to approach these models as tailored, focused examples of particular aspects of the disease, with each model offering advantages for studying a specific component. By synthesizing the findings of various models and comparing and contrasting the results, we can hopefully help define the full progression of DR and determine all the molecular and cellular components at play. Experimental models also hold a critical role for both developing and testing potential treatment options. It is important to have a sound understanding of the mechanisms of each model so as to understand what aspect of DR can be represented and studied. Without this understanding, researchers may be hindered by choosing unsuitable models or struggling with unrealistic expectations. This summary of what is currently known about some of the most popular experimental models of DR will hopefully help clarify some of the major differences between models and help determine which models are suitable for particular research aspects.

## Figures and Tables

**Table 1:** Summary of in vivo and in vitro models of diabetic retinopathy.

In Vivo Models					
Model	Type of Diabetes	Onset of Hyperglycemia	Main Retinal Outcomes	Advantages	Disadvantages
Streptozotocin (STZ)	Type 1	Within 1 week of injection	Retinal microglial cell activation [79]; retinal neovascularization [81]; increase in TNF-α, IL-1, IL-6, and VEGF expression [82]; functional abnormalities by ERG [80]	Low cost, readily available, easy to administer	Lack of ischemic phenotype; variation in phenotypes, in part due to disagreement over ideal protocol [88,89,90,91]; spontaneous β-cell regeneration may cause instability; significant amount of time required to reach moderate to severe phenotypes; mice present less severe retinal lesions compared to rats [125]
Alloxan	Type 1	Within 4–5 days of injection	Retinal pericyte ghosts and acellular capillaries [104]; functional abnormalities by ERG [174]	Low cost, readily available, easy to administer	Several notable inconsistencies and abnormal findings [102,103]; no consistent evidence of retinal vascular abnormalities [104,105,106]
Akita (Ins2^Akita^)	Type 1	Within 8 weeks	Increased retinal vascular permeability [110]; retinal thinning [110]; alteration in astrocytes and microglia [110]; RGC reduction [111]; functional abnormalities by ERG [109]	Commercially available with a variety of mouse genetic backgrounds, relatively consistent and predictable onset of phenotype	Does not allow for study of autoimmune process of DM [98]; β-cell regeneration is unlike human condition and can complicate study results [113]
Akimba (Ins2^Akita^/VEGF^+/−^)	Type 1	Within 8 weeks	Rod photoreceptor degeneration [115]; neovascularization [114]; capillary nonperfusion [114]; fibrosis [114]; edema [114]; increased pro-inflammatory and pro-angiogenic markers [119]	Higher blood glucose from earlier age [114]; consistent and predictable onset of phenotype; capable of replicating many phenotypical changes in DR	Vascular changes are not due to hyperglycemia but due to the transgene hVEGF_165_ [114]
Non-obese diabetic (NOD)	Type 1	12–30 weeks old (variation based on gender) [175]	Increase in VEGF [176]; increase in ganglion cell and endothelial cell apoptosis [176]; microaneurysms [124]; increase in retinal thickness [124]; vasculopathy and retinal edema [124]	Many similarities to human DR phenotype, polygenic model resulting in disease phenotype similar to human DM [177]	Requires specific pathogen-free environment due to propensity to immunomodulation [77]; varying incidence rate depending on gender [77,120]; more severe perinsulitis than humans [120,122]
Db/db	Type 2	4–8 weeks	Reduced retinal ganglion cells [125,126]; thinning of inner limiting membrane [126]; increased MMP-2 [123], MMP-2 [123], CD31 [126], VEGF [126], and HIF-1α [126]; reduced ERG amplitudes and ganglion cell loss [125,130]	Exhibits many features of neurodegenerative process seen in human eyes; hyperglycemia is main cause of neurodegenerative changes [125]	Neurodegenerative changes occur before any noted vascular changes are seen [130]
Goto–Kakizaki (GK)	Type 2	4–6 weeks	Retinal angiogenesis [132]; increase in VEGF, PDGF, MMP-2, MMP-9, and IGF-1 [132]; increased endothelial/pericyte ratio [133], alterations to photoreceptor outer segments and vacuolization of RPE cells [134]	Relatively consistent findings, commercially available, fasting glucose remains mild and stable throughout lifetime [77]	Early β cell destruction does not truly represent human type 2 diabetes [77]; phenotypes take significant time to develop
Pancreatectomy	Type 1	Varies depending on species	In cats: capillary basement membrane thickening [135]; microaneurysms [136]; capillary nonperfusion [136]; neovascularization [136] In monkeys: no consistent DR findings In dogs: no consistent DR findings	Useful for studying systemic Type 1 diabetic changes; confident and consistent induction of DM	Takes significant time to develop DR phenotype, with some species never developing DR phenotype; generally only performed on larger animals due to technical complexity
STZ + UCCAO	Type 1	Within 1 week of STZ injection	Increase in retinal inflammatory cells [155]; decrease in capillary vessel diameter [155]; increase in retinal mRNA expression of *Ccl2*, *Ccl12*, *Bnip3*, *Pdk1*, *Hsp25*, and *Vegfa* [155]	Includes ischemic phenotype in DM model; accelerates inflammatory and ischemic DR phenotype findings compared to STZ-only models	Requires some technical precision to perform UCCAO
**In Vitro Models**					
	**Advantages**			**Disadvantages**	
Cell monocultures	ARPE-19 cells useful for representing diseased-state RPE [158] fhRPE cells useful for studying barrier function of cells [158] Use of glial cells permits study of the response of Muller cells, astrocytes, and microglia to diabetic stress factors Stem cell-derived models allow for exploration of regenerative treatment options hiPSCs maintain physiological and morphological attributes of human RPE and exhibit similar functionality [170] Cell cultures are relatively low cost, with low resource requirements			Appropriate culturing conditions not fully described for all cell types [165] Cannot study interactions between cell types	
Cell co-cultures	All the benefits of cell monocultures Able to study interactions and signaling pathways between cell types Pericyte and endothelial cell cultures allow for studying diabetic retinal vascular damage [171] Microglia and endothelial cell cultures allow for studying influence of glial cells on hyperglycemia and DM drug efficacy [172]			Limited by the complexity of the model that can be explored	
3D cell models and organoids	Allow for complex study of mechanisms of DR without ethical constraints of in vivo models Retinal organoids can be used to study retinal and choroidal pathological vascular changes in DR [173]			Require more lab resources Require more technical skills	

## Data Availability

Not applicable as there is no newly generated data.

## Abbreviations

The following abbreviations are used in this manuscript:

DR	diabetic retinopathy
DM	diabetes mellitus
ROS	reactive oxygen species
AGEs	advanced glycation end products
PKC	protein kinase C
PEDF	pigment epithelium-derived factor
NADPH	nicotinamide adenine dinucleotide phosphate
DAG	diacylglycerol
PDR	proliferative diabetic retinopathy
NPDR	non-proliferative diabetic retinopathy
BRB	blood–retinal barrier
DME	diabetic macular edema
VEGF	vascular endothelial growth factor
HIF-1	hypoxia-inducible factor 1
IGF-1	insulin-like growth factor 1
STZ	streptozotocin
ICAM-1	intercellular adhesion molecule-1
TNF-α	tumor necrosis factor alpha
RAGE	receptor for advanced glycation end products
MCP-1:	monocyte chemoattractant protein-1
MMP-2	matrix metalloproteinase 2
MMP-9	matrix metalloproteinase 9
NOD	non-obese diabetic
UCCAO	unilateral common carotid artery occlusion
BCCAO	bilateral common carotid artery occlusion
OIS	ocular ischemic syndrome
HRMECs	human retinal vascular endothelial cells
AI	artificial intelligence
OCT	optical coherence tomography
fhRPE	fetal human RPE cells
hESCs	human embryonic stem cells
hiPSCs	human induced pluripotent stem cells
